# Gender Differences in the Associations Between Serum Urate and Cardiometabolic Risk Factors in Age-Matched Middle-Aged Japanese

**DOI:** 10.1177/26884844251379364

**Published:** 2025-09-17

**Authors:** Ichiro Wakabayashi, Takashi Daimon, Tetsuya Yamamoto

**Affiliations:** ^1^ Department of Preventive Medicine, School of Medicine, Hyogo Medical University, Nishinomiya, Hyogo, Japan.; ^2^ Department of Biostatistics, School of Medicine, Hyogo Medical University, Nishinomiya, Hyogo, Japan.; ^3^ Department of Health Evaluation Center, Osaka Gyoumeikan Hospital, Osaka, Japan.

**Keywords:** cardiovascular risk factor, cutoff value, gender difference, metabolic syndrome, serum urate

## Abstract

**Background:**

The purpose of this study was to determine whether gender affects associations between serum urate and cardiometabolic risk factors in age-matched men and women.

**Methods:**

The subjects were 4612 men and 4612 age-matched women (35–60 years old) who had undergone health-checkup examinations. The relationships between high urate (defined as the highest quartile for serum urate) and cardiovascular risk factors were compared between men and women using logistic regression analysis and receiver operating characteristic analysis.

**Results:**

The odds ratio (OR) of subjects with high urate versus subjects without high urate for metabolic syndrome was significantly higher in women than in men (women: 3.82 [95% confidence interval: 2.93–4.97] vs. men: 1.99 [1.63–2.42], *p* < 0.01), and the ORs for each of the cardiometabolic risk factors (high body mass index [BMI], hypertension, hypertriglyceridemia, low HDL cholesterolemia, and diabetes mellitus) were also significantly higher in women than in men. The ORs of the interaction term consisting of gender (women vs. men) and high urate for each of the cardiometabolic risk factors (high BMI, hypertriglyceridemia, low HDL cholesterolemia, and diabetes mellitus) and metabolic syndrome were significantly higher than the reference level. In receiver operating characteristic analysis, area under the curve of the relationship between serum urate and each of high BMI, hypertension, hypertriglyceridemia, low HDL cholesterolemia, high LDL cholesterolemia, diabetes, and metabolic syndrome was significantly larger in women than in men. The cutoffs of serum urate for metabolic syndrome were 6.25 and 4.65 mg/dL in men and women, respectively, which are lower than those currently used.

**Conclusions:**

The associations between hyperuricemia and cardiometabolic risk factors were age-independently stronger in women than in men. The results suggested that the cutoff values for hyperuricemia that are currently used should be redefined from the viewpoint of prevention of cardiovascular disease.

## Introduction

Serum urate is known to be associated with the risk of cardiovascular disease,^1,2^ although their causal relationship remains to be clarified and urate is not included in the criteria of metabolic syndrome. Pathological consequences of uric acid have been suggested in elevation of blood pressure and progression of atherosclerosis through endothelial dysfunction due to uric acid-induced increases in oxidative stress and pro-inflammatory cytokines.^3^ However, it might be difficult to prove causality of the relationship between uric acid and atherosclerotic disease because of confounding by a variety of factors, including obesity, hypertension, diabetes mellitus, metabolic syndrome, and alcohol consumption.

There is a gender difference in blood urate levels, which is mainly explained by estrogen that enhances urinary clearance of urate.^4,5^ It has been reported that there is also a gender difference in the relationship between serum urate and risk of cardiovascular disease: Serum urate was reported to be an independent predictor of cardiovascular death in men but not in women,^6^ and serum urate showed a significant positive correlation with Framingham risk score in men but not in women.^7^ In contrast, the associations of serum urate with metabolic syndrome and its components have been shown to be stronger in women than in men.^8–11^ Moreover, serum urate showed a significant positive association with arterial stiffness in women but not in men in a general population and in hypertensive patients with chronic kidney disease.^12–14^ Thus, there are dissociations in the previous studies regarding the gender differences in the associations between serum urate and cardiovascular risk. Age as well as gender is known to influence the relationships between serum urate level and cardiovascular risk factors.^8,15–21^ However, to the best of our knowledge, there has been no report showing gender differences in the relationships between serum urate and cardiovascular risk factors in an age-matched cohort.

The cutoff values (7 mg/dL for men and 6–7 mg/dL for women) of serum urate levels for development of gout disease are generally used in health checkups, but they have recently been shown to be different from the cutoff values of serum urate for predicting cardiovascular disease, which were lower than the cutoff values for gout: In a recent follow-up study, the URRAH (Uric Acid Right for Heart Health) study, in Italy, the cutoff values for predicting myocardial infarction were 5.49 mg/dL in men and 5.26 mg/dL in women^22^; the cutoff values for predicting all cases and fatal cases of heart failure were 5.34 and 4.89 mg/dL, respectively, for men and women together^23^; and the cutoff value for predicting cerebrovascular events was 4.79 mg/dL for men and women together.^24^ In a follow-up study in China, the cutoff values of serum urate for metabolic syndrome were 6.3 mg/dL in men and 4.9 mg/dL in women.^25^ Thus, the cutoff values of serum urate clearly differ for gout and cardiovascular diseases, and redefinition of the cutoffs of serum urate is thought to be needed for early prevention of cardiovascular disease. Moreover, the cutoff values of serum urate for starting to precipitate in synovial fluid have recently been reported to be 4.95 mg/dL in men and 3.9 mg/dL in women,^26^ which are also much lower than the cutoffs for gout.

The purpose of this study was therefore to investigate differences in relationships between blood urate level and cardiometabolic risk factors in age-matched men and women and to determine the cutoff values of urate from the viewpoint of discrimination of cardiometabolic risk. Since age was reported to considerably affect relationships between serum urate and cardiometabolic risk factors,^8,15–21^ we prepared subject groups of men and women whose ages were completely matched for investigating age-independent gender differences in the above relationships using two statistical analyses, logistic regression analysis and receiver operating characteristic (ROC) analysis.

## Methods

### Subjects

The subjects were Japanese men (*n* = 4612) and age-matched women (*n* = 4612) aged 35–60 years (median: 45 years) who had received periodic health-checkup examinations at workplaces in a district of Japan from April 2005 to March 2006. Data for the original database used in this study were obtained in annual health-checkup examinations for workers in Yamagata Prefecture in Japan. The protocol of this study was approved by the Hyogo College of Medicine Ethics Committee (No. 3003 in 2018). Since the number of male subjects was larger than the number of women at all of the above ages in the original database, male subjects of the same number as that of female subjects at each age were randomly selected from the original database using random digits. A cross-sectional study was performed using the above local population-based database of workers.

### Questionnaires on lifestyles and illnesses of subjects

Information on histories of alcohol consumption, cigarette smoking, regular exercise, illness, and therapy for illness was obtained using self-reported questionnaires at the health checkups. The questionnaires include simple standard questions on individual lifestyles and histories of disease and medication therapy as follows.

History of medication therapy was simply asked in the questionnaire for each of the diseases, including hypertension, dyslipidemia, hyperuricemia, and diabetes as “Are you receiving medication therapy to lower blood pressure level,” “are you receiving medication therapy to lower blood cholesterol,” “are you receiving medication therapy to lower blood urate level,” and “are you receiving insulin injection or medication therapy to lower blood sugar level?” respectively. Subjects receiving medication therapy to lower serum urate level were excluded from the subjects for analysis.

Average alcohol consumption per week of each subject was reported on the questionnaires. Frequency of habitual alcohol drinking was asked in the questionnaire as “How frequently do you drink alcohol?” Frequency of weekly alcohol drinking was categorized as “every day” (regular drinkers), “sometimes” (occasional drinkers), and “never” (nondrinkers). Cigarette smokers were defined as subjects who had smoked for 6 months or longer and had smoked for the past month or longer. Then the subjects who were smokers were further asked “What is your average cigarette consumption per day?”. The response categories for this question were “less than 21 cigarettes per day,” “21 or more and less than 41 cigarettes per day,” and “41 or more cigarettes per day.” Because the proportion of subjects with daily cigarette consumption of 41 or more cigarettes was very low (men, *n* = 17; women, *n* = 1), the subjects were divided into three categories of nonsmokers, light smokers (20 or less cigarettes per day), and heavy smokers (21 or more cigarettes per day). Subjects with a habit of regular exercise were defined as those doing exercise almost every day for 30 min or longer per day.

### Measurements

Height and body weight were measured with the subjects wearing light clothes at the health checkup. Body mass index (BMI) was calculated as weight in kilograms divided by the square of height in meters. Waist circumference was measured at the navel level according to the recommendation of the definition of the Japanese Committee for the Diagnostic Criteria of Metabolic Syndrome,^27^ and visceral obesity was evaluated by the ratio of waist circumference (cm) to height (cm) (waist-to-height ratio: WHtR). Blood pressure was measured by trained nurses, who were part of the local health-checkup company, with a mercury sphygmomanometer once on the day of the health checkup after each subject had rested quietly in a sitting position. Korotkoff phase V was used to define diastolic pressure. Fasted blood was sampled from each subject in the morning, and serum urate, triglycerides, HDL cholesterol, and LDL cholesterol were measured by enzymatic methods using commercial kits, namely, Pureauto S UA, Pureauto S TG-N, Cholestest N-HDL, and Cholestest LDL (Sekisui Medical Co., Ltd, Tokyo, Japan), respectively. Hemoglobin A_1c_ was measured by the NGSP (National Glycohemoglobin Standardization Program)-approved technique using the latex cohesion method with a commercial kit (Determiner HbA_1c_, Kyowa Medex, Tokyo, Japan). Since the standards of hemoglobin A_1c_ used for measurement are different in the NGSP method and JDS (Japan Diabetes Society) method, hemoglobin A_1c_ values were calibrated using a formula proposed by the JDS: hemoglobin A_1c_ (NGSP) (%) = 1.02 × hemoglobin A_1c_ (JDS) (%) + 0.25 (%).^28^ Coefficients of variation for reproducibility of each measurement were ≤3% for urate and triglycerides and were ≤5% for HDL cholesterol, LDL cholesterol, and hemoglobin A_1c_. Cardiometabolic index (CMI) was calculated as a product of the ratio of waist circumference (cm) to height (cm) and the ratio of triglycerides (mg/dL) to HDL cholesterol (mg/dL). The cutoff values for CMI used were 1.625 for men and 0.800 for women.^29^

### Criteria of metabolic syndrome

Metabolic syndrome was defined, according to the criteria by IDF (International Diabetes Federation)^30^ with a slight modification, as the presence of two or more risk factors in addition to visceral obesity diagnosed as high WHtR. Risk factors included in the criteria are visceral obesity, high blood pressure, dyslipidemia (low HDL cholesterol and/or high triglycerides), and hyperglycemia evaluated by hemoglobin A_1c_. The criterion for each risk factor was defined as follows: visceral obesity, WHtR ≥0.5^31^; hypertension, systolic blood pressure ≥140 mmHg and/or diastolic blood pressure ≥90 mmHg^32^; high triglycerides, triglycerides ≥150 mg/dL; low HDL cholesterol, HDL cholesterol <40 mg/dL; high LDL cholesterol, ≥140 mg/dL^33,34^; and diabetes, hemoglobin A_1c_ ≥ 6.5%.^35^ Subjects receiving drug therapy for hypertension, dyslipidemia, and diabetes were also included in the above definitions of hypertension, dyslipidemia, and diabetes, respectively.

### Statistical analysis

The values of serum urate in the subjects were arranged in ascending order, and then the subjects were divided into four quartile groups of almost equal sizes. Serum urate concentrations were measured as a unit of mg/dL with one decimal place, and there was a considerable number of subjects who showed the same value of urate concentration at each quartile border. Thus, it was impossible to divide the subjects into four quartile groups consisting of completely equal numbers of subjects. The subjects were therefore divided into four quartile groups with similar numbers of subjects: 1111 men and 1070 women in the 1st quartiles, 1181 men and 1207 women in the 2nd quartiles, 1130 men and 1157 women in the 3rd quartiles, and 1190 men and 1178 women in the 4th quartiles. Subjects with high serum urate were defined as subjects in the highest (4th) groups of urate in men and women. When the currently used cutoff of serum urate level (7 mg/dL for men and women) was used, frequency of hyperuricemia was much lower in women than in men (21.4% [*n* = 988] vs. 0.74% [*n* = 34]), which should affect results of comparison between men and women. Therefore, we used the above definition of high urate (highest quartile), providing similar proportions of subjects with high urate in men and women. Each variable was compared between men and women by analyses as mentioned below. Categorical variables were compared by means of Pearson’s chi-square test for independence. Continuous variables were presented as means with standard deviations and compared using unpaired Student’s *t* test in parametric analysis and were presented as median levels with interquartile ranges and compared using Mann–Whitney’s *U* test in nonparametric analysis. Triglyceride levels are known not to show a normal distribution, and triglycerides and CMI were therefore presented as medians with 25 and 75 percentile values and were compared among the groups nonparametrically. In logistic regression analysis, crude and adjusted odds ratios (ORs) for each cardiometabolic risk factor were estimated. Crude ORs were compared between men and women using the Breslow–Day test. In multivariable logistic regression analysis, age and habits of smoking, alcohol consumption, and regular exercise were used as other explanatory variables. ORs for each risk factor of the interaction term consisting of gender (women vs. men) and high urate (highest quartile vs. other quartiles) were also estimated with adjustment for the above variables. ROC analysis was performed to investigate relationships between urate and cardiometabolic risk factors. The area under the ROC curve (AUC) with 95% confidence interval was estimated empirically and compared between men and women using the bootstrap test. Optimal cutoff points of serum urate for men and women were obtained using metabolic syndrome as an outcome. Each optimal cutoff point was selected by maximizing Youden’s index, which is the difference between the true positive rate (sensitivity) and the false positive rate (1-specificity) in the ROC curve. Probability (*p*) values less than 0.05 were defined as significant. Statistical analyses were performed using computer software programs (SPSS version 25.0 for Windows, IBM Corp., Armonk NY, USA; R version 4.0.3).^36^

## Results

### Comparisons of variables related to cardiovascular disease in men and women

Table 1 shows the characteristics of the subjects regarding cardiovascular risk factors, including serum urate in men and women. The proportions of light smokers, heavy smokers, and regular drinkers were significantly higher in men than in women. BMI, systolic and diastolic blood pressure, urate, triglycerides, LDL cholesterol, hemoglobin A_1c,_ and CMI were significantly higher in men than in women, and HDL cholesterol was significantly lower in men than in women. The proportions of subjects with high BMI, hypertension, high urate, high triglycerides, low HDL cholesterol, high LDL cholesterol, dyslipidemia, diabetes mellitus, and metabolic syndrome were significantly higher in men than in women, and the proportions of subjects with histories of therapy for hypertension, dyslipidemia, and diabetes were also significantly higher in men than in women.

**Table 1.** Characteristics of the Male and Female Subject Groups

Variables	Men (*n* = 4612)	Women (*n* = 4612)
Age (years)	45 (40, 52)	45 (40, 52)
Smokers (%): light; heavy	42.3; 15.5	19.6**; 0.7**
Drinkers (%): occasional; regular	35.2; 45.3	33.0; 10.1**
Regular exercise (%)	11.0	5.4**
Height (cm)	170.6 ± 6.0	157.4 ± 5.5**
Body weight (Kg)	68.5 ± 10.8	54.5 ± 9.1**
BMI (kg/m^2^)	23.52 ± 3.33	21.98 ± 3.50**
High BMI (%)	28.5	16.8**
Waist circumference (cm)	83.2 ± 8.9	76.6 ± 9.6**
Waist-to-height ratio	0.488 ± 0.052	0.487 ± 0.063
High waist-to-height ratio (%)	39.2	37.8
Systolic blood pressure (mmHg)	125.7 ± 16.0	118.0 ± 16.9**
Diastolic blood pressure (mmHg)	77.5 ± 11.3	70.7 ± 11.2**
Hypertension (%)	26.7	15.8**
Therapy for hypertension (%)	10.3	7.1**
Urate (mg/dL)	6.00 ± 1.29	4.21 ± 0.94**
High urate (%)	21.4	0.74**
Triglycerides (mg/dL)	107 (72, 160)	65 (47, 92)**
High triglycerides (%)	28.8	6.9**
HDL cholesterol (mg/dL)	56.6 ± 14.7	65.8 ± 14.7**
Low HDL cholesterol (%)	9.5	2.1**
LDL cholesterol (mg/dL)	118.6 ± 30.8	112.9 ± 29.9**
High LDL cholesterol (%)	24.1	17.7**
Dyslipidemia (%)	34.2	11.3**
Therapy for dyslipidemia (%)	4.3	3.9
Hemoglobin A_1C_ (%)	5.42 ± 0.67	5.33 ± 0.45**
Diabetes mellitus (%)	5.6	2.1**
CMI	0.948 (0.555, 1.676)	0.472 (0.309, 0.789)**
High CMI (%)	26.1	24.5
Therapy for diabetes mellitus (%)	3.3	1.3**
Metabolic syndrome (%)	10.2	5.2**

Shown are proportions, means with standard deviations, or medians with interquartile ranges of each variable. Asterisks indicate significant differences from men (**p < 0.01).

BMI, body mass index; CMI, cardiometabolic index.

### Distributions of serum urate levels in men and women

**FIG. 1.**
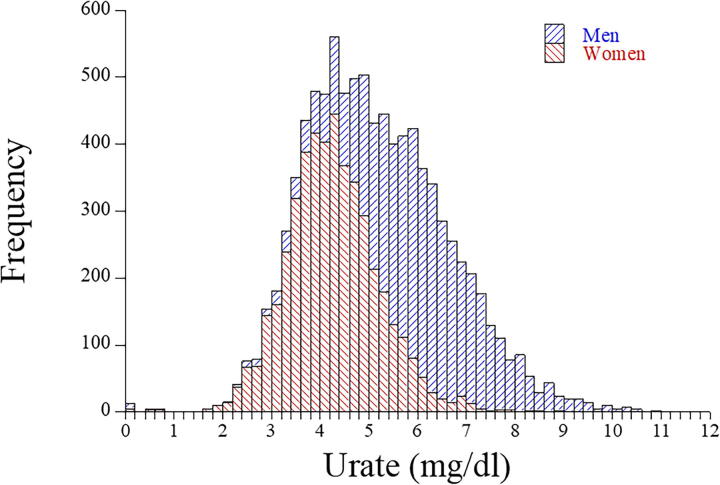
A histogram for serum urate levels in men and women.

A histogram of urate levels in men and women is shown in Figure 1. Urate values in men were distributed at higher levels than those in women.

### Comparisons of prevalences of each cardiovascular risk factor in the subject groups with and without high serum urate in men and women

Table 2 shows the prevalences of various cardiovascular risk factors in the high (4th quartile) and not-high (1st, 2nd, or 3rd quartile) serum urate groups of men and women. Subjects with high serum urate were defined as those in the highest quartile for serum urate. Using this definition of high serum urate, the proportions of subjects having each cardiometabolic risk factor were compared between high and not-high urate groups and between men and women. In men and women, the prevalences of all of the cardiovascular risk factors tested (high BMI, high WHtR, hypertension, high triglycerides, low HDL cholesterol, high LDL cholesterol, diabetes mellitus, high CMI, and metabolic syndrome) were significantly higher in the subject group with high urate than in the group without high urate in both men and women except for the prevalence of diabetes mellitus in men, which was significantly higher in the subject group without high urate than in the group with high urate. Both in the groups with and without high serum urate, the prevalences of high BMI, hypertension, high triglycerides, low HDL cholesterol, high LDL cholesterol, and metabolic syndrome were significantly higher in men than in women, whereas the prevalence of high WHtR was not significantly different between men and women. The prevalences of diabetes mellitus and high CMI were significantly higher in men than in women in the group without high urate, but not in the group with high urate.

**Table 2.** Prevalences of Cardiometabolic Risk Factors in Different Groups Divided by Serum Urate Levels in Men and Women

Cardiometabolic risk factors	Men	Women
High BMI (%)		
1st–3rd vs. 4th quartile groups of urate	24.2 vs. 41.0**	11.8^††^ vs. 31.4**^,††^
High waist-to-height ratio (%)		
1st–3rd vs. 4th quartile groups of urate	33.9 vs. 54.4**	32.1 vs. 54.3**
Hypertension (%)		
1st–3rd vs. 4th quartile groups of urate	23.1 vs. 37.2**	12.0^††^ vs. 27.1**^,††^
High triglycerides (%)		
1st–3rd vs. 4th quartile groups of urate	24.5 vs. 41.2**	4.6^††^ vs. 13.8**^,††^
Low HDL cholesterol (%)		
1st–3rd vs. 4th quartile groups of urate	8.6 vs. 12.0**	1.5^††^ vs. 4.2**^,††^
High LDL cholesterol (%)		
1st–3rd vs. 4th quartile groups of urate	21.9 vs. 30.5**	15.4^††^ vs. 24.2**^,††^
Diabetes (%)		
1st–3rd vs. 4th quartile groups of urate	6.1 vs. 4.2*	1.7^††^ vs. 3.3**
High CMI (%)		
1st–3rd vs. 4th quartile groups of urate	21.9 vs. 38.4**	19.7^†^ vs. 38.5**
Metabolic syndrome (%)		
1st–3rd vs. 4th quartile groups of urate	8.4 vs. 15.4**	3.2^††^ vs. 11.1**^,††^

Shown are proportions of each cardiometabolic risk factor. Symbols indicate significant differences from the 1st, 2nd, and 3rd quartile groups of serum urate (*p < 0.05; **p < 0.01) and men (^†^*p* < 0.05; ^††^*p* < 0.01).

### Comparison between men and women of ORs of the 4th quartile versus the other quartile groups for each cardiometabolic risk factor

ORs for each cardiometabolic risk factor of the subject group with high serum urate (4th quartile for urate) versus the subject group without high serum urate (1st, 2nd, and 3rd quartiles for urate) were compared between men and women (Table 3). In both men and women, the ORs for cardiometabolic risk factors of the subject group with high urate (4th quartile for urate) versus the subject group without high urate were significantly higher than the reference level of 1.00 except for the OR for diabetes in men, which was significantly lower than the reference level. In univariable analysis, the ORs for high BMI, hypertension, high triglycerides, low HDL cholesterol, diabetes mellitus, and metabolic syndrome were significantly higher in women than in men. The ORs of the interaction term consisting of gender (women vs. men) and high serum urate (4th vs. 1st, 2nd, and 3rd quartiles for serum urate) for high BMI, high triglycerides, low HDL cholesterol, diabetes mellitus, and metabolic syndrome were significantly higher than the reference level, whereas the ORs of the interaction term for high WHtR, hypertension, high LDL cholesterol, and high CMI were not significantly different from the reference level.

**Table 3.** Odds Ratios for Cardiometabolic Risk Factors of Subjects of the 4th Quartile Versus the Other Quartiles for Serum Urate Levels and the Interaction Term Consisting of Gender and High Urate (Subjects in the 4th Quartile Group of Serum Urate Levels)

Cardiometabolic risk factors	Univariable	Multivariable
OR for high BMI		
Men	2.18 (1.90–2.51)**	2.31 (2.01–2.67)**
Women	3.43 (2.92–4.03)**^,††^	3.53 (3.00–4.17)**
Interaction term	—	1.53 (1.23–1.89)**
OR for high waist-to-height ratio		
Men	2.32 (2.03–2.65)**	2.47 (2.15–2.84)**
Women	2.51 (2.20–2.88)**	2.45 (2.13–2.83)**
Interaction term	—	0.98 (0.81–1.19)
OR for hypertension		
Men	1.98 (1.72–2.28)**	2.10 (1.80–2.44)**
Women	2.73 (2.32–3.22)**^,††^	2.48 (2.08–2.96)**
Interaction term	—	1.11 (0.89–1.40)
OR for high triglycerides		
Men	2.16 (1.88–2.48)**	2.16 (1.88–2.49)**
Women	3.33 (2.64–4.19)**^,††^	3.06 (2.42–3.88)**
Interaction term	—	1.42 (1.08–1.87)*
OR for low HDL cholesterol		
Men	1.46 (1.18–1.80)**	1.73 (1.39–2.16)**
Women	2.94 (1.97–4.38)**^,††^	3.09 (2.05–4.64)**
Interaction term	—	1.81 (1.15–2.86)*
OR for high LDL cholesterol		
Men	1.57 (1.35–1.82)**	1.68 (1.45–1.96)**
Women	1.75 (1.49–2.06)**	1.62 (1.36–1.92)**
Interaction term	—	1.00 (0.80–1.25)
OR for diabetes		
Men	0.67 (0.49–0.92)*	0.72 (0.52–0.99)*
Women	1.93 (1.28–2.90)**^,††^	1.67 (1.10–2.54)*
Interaction term	—	2.28 (1.35–3.84)**
OR for high CMI		
Men	2.23 (1.93–2.57)**	2.30 (1.99–2.66)**
Women	2.55 (2.21–2.95)**	2.49 (2.14–2.90)**
Interaction term	—	1.03 (0.84–1.27)
OR for metabolic syndrome		
Men	1.99 (1.63–2.42)**	2.12 (1.73–2.60)**
Women	3.82 (2.93–4.97)**^,††^	3.48 (2.65–4.56)**
Interaction term	—	1.57 (1.12–2.21)**

Shown are odds ratios (ORs) for high BMI, high waist-to-height ratio, hypertension, high triglycerides, low HDL cholesterol, high LDL cholesterol, diabetes mellitus, high CMI, and metabolic syndrome with their 95% confidence intervals. In multivariable analysis, age and habits of smoking, alcohol consumption, and regular exercise were adjusted. In addition, medication therapy for dyslipidemia was adjusted in analyses for high triglycerides, low HDL cholesterol, high LDL cholesterol, and CMI. The interaction term consisted of gender (women vs. men) and high serum urate (4th vs. 1st, 2nd, and 3rd quartiles for serum urate). Symbols indicate significant differences from the reference level (*p < 0.05; *p < 0.01) and from men (^††^*p* < 0.01).

OR, odds ratio.

### Comparison between men and women of AUC for the relationship of urate with each cardiometabolic risk factor in ROC analysis

AUCs for the relationship between urate and each cardiometabolic risk factor were compared in men and women (Table 4). AUCs for the relationships of urate with high BMI, hypertension, high triglycerides, low HDL cholesterol, high LDL cholesterol, diabetes, and metabolic syndrome were significantly larger in women than in men, whereas there were no significant differences in AUCs for the relationships of urate with high WHtR and high CMI.

**Table 4.** Comparisons of Area Under the Receiver Operating Characteristic Curve for the Relationships between Serum Urate Levels and Each Cardiometabolic Risk Factor in the Male and Female Subject Groups

Cardiometabolic risk factor	Men	Women
High BMI	0.625 (0.607–0.643)	0.679 (0.658–0.701)**
High waist-to-height ratio	0.625 (0.608–0.641)	0.637 (0.621–0.654)
Hypertension	0.594 (0.575–0.613)	0.646 (0.624–0.669)**
High triglycerides	0.620 (0.602–0.638)	0.694 (0.662–0.725)**
Low HDL cholesterol	0.581 (0.554–0.608)	0.644 (0.587–0.701)*
High LDL cholesterol	0.566 (0.547–0.585)	0.605 (0.584–0.627)**
Diabetes mellitus	0.420 (0.384–0.457)	0.605 (0.552–0.658)**
High CMI	0.629 (0.611–0.647)	0.647 (0.628–0.666)
Metabolic syndrome	0.605 (0.578–0.632)	0.710 (0.675–0.745)**

Shown are AUCs with 95% confidence intervals of each cardiometabolic risk factor in relation to serum urate levels. Asterisks indicate significant differences from men (*p < 0.05; **p < 0.01). AUC, area under the receiver operating characteristic curve.

### ROC curves of the relationships between urate and metabolic syndrome in men and women

**FIG. 2.**
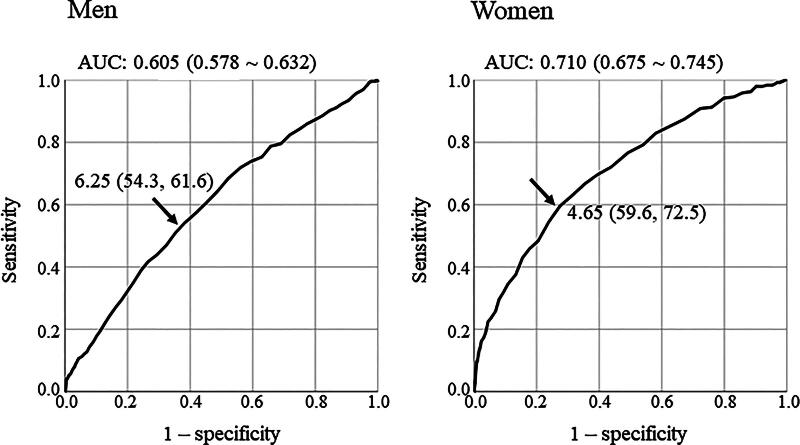
Receiver operating characteristic curves for the relationships between serum urate levels and metabolic syndrome in men and women. Cutoff points are indicated by the arrows, and cutoff values (mg/dL) with their sensitivity and specificity values (%) are shown in the figures. Areas under the ROC curves (AUCs) and their 95% confidence intervals are also shown in the figures. ROC, receiver operating characteristic.

Figure 2 shows ROC curves for the relationships between urate and metabolic syndrome in men and women. The cutoff values for metabolic syndrome with their sensitivity and specificity were 6.25 mg/dL (54.3% and 61.6%) in men and 4.65 mg/dL (59.6% and 72.5%) in women. When the above cutoff values were used, the proportions of subjects showing high serum urate levels were 40.0% in men and 29.2% in women, and the ORs with 95% confidence intervals for metabolic syndrome of subjects with high serum urate versus subjects without high serum urate were 2.04 (1.67–2.48) in men and 3.48 (2.65–4.58) in women in multivariable logistic regression analysis with adjustment for age and habits of smoking, alcohol consumption, and regular exercise. The OR for metabolic syndrome of the interaction term consisting of gender (women vs. men) and high urate was 1.65 [1.18–2.31] in multivariable analysis, which was significantly higher than the reference level (*p* < 0.01) and was similar to OR of the interaction term consisting of gender and high urate defined as the highest quartile group of urate (1.57 [1.12–2.21]) (Table 3).

### Proportions of subjects with high urate levels defined using different cutoff values

Proportions of subjects with high urate levels defined using the cutoff value of 6.0 or 7.0 mg/dL were as follows: urate 6.0 mg/dL: 50.3% in men and 3.7% in women; urate 7.0 mg/dL: 21.4% in men and 0.7% in women. When the cutoff values obtained in the present study were used, the percentages of subjects with high urate were 40.0% in men and 29.2% in women. Thus, the percentages of subjects with high urate were very low in women when using the cutoff value of 6.0 or 7.0 mg/dL.

### Comparison between men and women of the prevalences of metabolic syndrome defined using different criteria

**FIG. 3.**
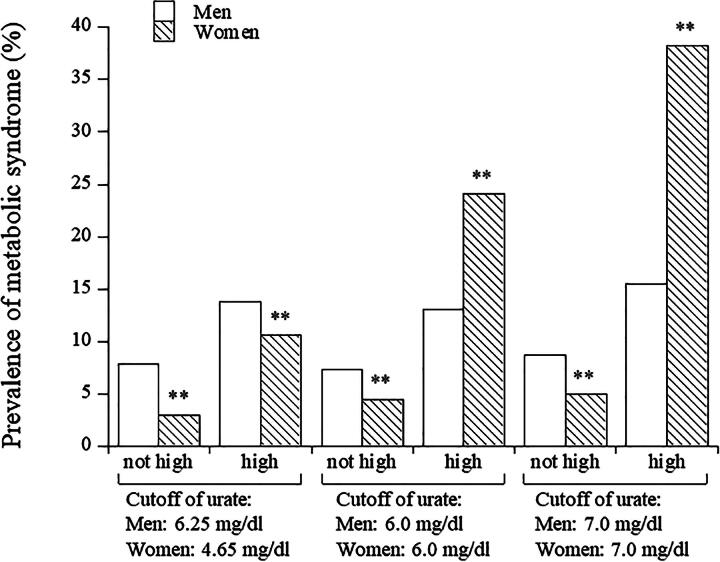
Comparisons of prevalences of metabolic syndrome between men and women with and without high urate. High urate was defined using different cutoffs. Asterisks denote significant differences from men (**p < 0.01).

The prevalences of metabolic syndrome were compared in men and women when dividing the population into two groups with and without high urate using different cutoffs of serum urate level of 6.0 mg/dL, 7.0 mg/dL, and the values obtained by ROC analysis in this study (men, 6.25 mg/dL; women, 4.65 mg/dL). As shown in Figure 3, in analysis using the cutoffs of 6.0 or 7.0 mg/dL in men and women, the prevalence of metabolic syndrome in the group without high urate levels was significantly higher in men than in women, whereas the prevalence in the group of subjects showing high urate levels was significantly higher in women than in men. When the above cutoffs obtained in the present study were used for analysis, the prevalences of metabolic syndrome both in the groups with and without high urate levels were significantly higher in men than in women.

## Discussion

This study demonstrated that there is a gender-specific difference in the degree of the association between blood urate level and cardiometabolic risk using logistic regression analysis and ROC analysis in age-matched middle-aged men and women. This gender difference did not depend on age, but depended on cardiometabolic risk factors: The associations of urate with high BMI, hypertension, high triglycerides, low HDL cholesterol, high LDL cholesterol, and diabetes mellitus were stronger in women than in men, whereas the degrees of the associations with high WHtR and CMI, which also reflects WHtR, were comparable in men and women. Consequently, the associations between serum urate and metabolic syndrome, for which criteria include hypertension, high triglycerides, low HDL cholesterol, and diabetes, were stronger in women than in men (OR for metabolic syndrome [4th vs. 1st, 2nd, and 3rd quartiles for urate]: 1.99 in men versus 3.82 in women [*p* < 0.01]: AUC for the relationship between urate and metabolic syndrome: 0.605 in men vs. 0.710 in women [*p* < 0.01]). This is, to the best of our knowledge, the first study that showed a gender difference in the association between urate and cardiometabolic risk factors in an age-matched cohort of men and women.

In a recent cross-sectional study in Japan, the ORs of hyperuricemia for cardiometabolic risk factors, including abdominal obesity, hyperglycemia, hypertriglyceridemia, low HDL cholesterolemia, and hypertension, were 1.3–2.5 times higher in women than in men.^11^ These findings except for the results for abdominal obesity and hypertension agree with the findings of the present study shown in Table 3 (ORs of the interaction term: 1.42–2.28). Although the associations between BMI and serum urate were stronger in women than in men in both logistic regression analysis and ROC analysis, the association between serum urate and abdominal obesity, evaluated by WHtR, was comparable in men and women in the present study. This dissociation regarding gender and the association between urate and abdominal obesity may be due to a difference in the indicators of abdominal obesity used: Waist circumference with different criteria of abdominal obesity for men and women (men, 85 cm; women, 90 cm) was used in the above previous study,^11^ whereas we used WHtR with the same cutoff value of 0.5 for men and women. WHtR is an indicator of abdominal obesity corrected by height and was suggested to be more suitable than waist circumference alone for evaluation of abdominal obesity.^31,37^ In fact, the associations between serum urate and waist circumference were significantly stronger in women than in men in logistic regression analysis and ROC analysis in the present study (data not shown). The reason for the above dissociation of the results for the relationships of serum urate with WHtR and waist circumstance remains unknown. WHtR is included in the components of CMI, and thus, no significant gender difference in the association between urate and CMI may reflect the above finding for WHtR. The reason for the dissociation in the gender difference or not in the associations of serum urate with BMI and WHtR also remains to be clarified. Regarding the relationships between serum urate and diabetes, a positive association and an inverse association were shown in women and men, respectively (Tables 3 and 4). Renal tubular reabsorption of urate is increased by insulin.^38,39^ Therefore, the inverse association in men may be explained by increased excretion of urate due to deficiency of insulin action in individuals with diabetes mellitus, of which the proportion was much higher in male subjects than in female subjects in the present study (Table 1).

In this study, a gender difference in the association between serum urate and hypertension was shown in univariable logistic regression analysis and ROC analysis, but not in multivariable logistic regression analysis. This dissociation may be caused by confounding factors (age and lifestyles) for the association since no adjustment is performed in ROC analysis as well as in univariable logistic regression analysis. Regarding the association between urate and LDL cholesterol, a gender difference was shown in ROC analysis, but not in univariable or multivariable logistic regression analysis. Results of ROC analysis reflect relationships of serum urate as a continuous variable, whereas results of logistic regression analysis reflect relationships of serum urate as a binary variable. Therefore, distributions of urate levels in men and women might affect the results for the gender difference in the association between urate and LDL cholesterol.

In the ROC analysis, we determined the cutoff values of serum urate using metabolic syndrome as an outcome. The cutoff values were 6.25 mg/dL in men and 4.65 mg/dL in women. These cutoffs are similar to the cutoffs for metabolic syndrome in men and women determined in a previous follow-up study in China (6.3 vs. 4.9 mg/dL).^25^ These values are clearly lower than the cutoff values of urate (7 mg/dL for men and 6–7 mg/dL for women) that were determined by the risk of crystallization of uric acid in tissues and are generally used at health checkups. The findings of the present study indicate that when the conventional cutoff value of 6.0 or 7.0 mg/dL was used for the definition of high urate, the percentage of subjects with high urate was extremely low in women, whereas the prevalence of metabolic syndrome was extremely high in women with high urate (Fig. 3). Thus, both in men and women, the cutoff concentrations of serum urate for prevention of cardiovascular disease are thought to be lower than that for prevention of diseases caused by uric acid crystals, for example, gout and gouty kidney. The associations between serum urate and cardiometabolic risk factors are stronger in women than in men, suggesting the possibility that serum urate is a more robust predictor of cardiovascular disease in women than in men. Therefore, the currently used cutoffs for hyperuricemia, especially in women, are obviously too low from the viewpoint of cardiovascular health and should be reconsidered. If our hypothesis on the thresholds of serum urate with a gender difference in the present study is correct, there must be a mechanism by which urate dissolved in the blood directly causes vascular damage. One possibility for this mechanism is that urate at even low concentrations around 4.6 mg/dL in the blood can cause endothelial dysfunction in women through increasing oxidative stress and pro-inflammatory cytokines.^3^ This hypothesis needs to be tested by experimental studies in the future. The thresholds for the 4th (highest) quartiles of men and women were 6.8 and 4.8 mg/dL, respectively. Thus, the threshold for the 4th quartile of women was close to the cutoff value (4.65 mg/dL) obtained in ROC analysis in the present study.

There are limitations of this study. The subjects were middle-aged Japanese men and women, and thus, further studies using cohorts of different ages and ethnicities, which were reported to affect serum urate level,^8,40^ are needed to confirm the findings of the present study. In fact, ethnic differences were suggested regarding the relationship between serum urate level and metabolic syndrome in previous studies.^41,42^ Since the subjects of this study were workers at companies in Yamagata, a local district in Japan, there is a possibility of a bias caused by healthy worker effect. In addition, the cohort of this study does not completely represent the population of Japan. The AUC for the relationship between urate and metabolic syndrome was not large in men, and thus, further studies are needed to determine more accurate cutoff of serum urate in men. Habitual alcohol drinking influences serum urate level and was thus used as an explanatory variable in multivariable analysis. However, subjects were classified into only three groups by weekly frequency of drinking. The influence of alcohol drinking on serum urate differs depending on the amounts of purines included in various alcohol beverages,^43,44^ for which information was not included in the database used in the present study. Information on menopausal status, which affects serum urate level,^45,46^ was also unavailable in this study. Furthermore, the database used did not include information on other possible confounding factors such as foods, nutrition, and socioeconomic status, for the relationships between serum urate level and cardiometabolic risk factors. Measurements of blood examinations related to atherosclerotic risk factors, including urate for all of the subjects, were performed at the same laboratory. Thus, there is a relatively low possibility of dilution bias in the present study. Since this study was a cross-sectional study, further prospective studies are needed to discuss causality of serum urate and cardiovascular risk.

## Conclusions

Women showed stronger age-independent associations than did men between hyperuricemia and cardiometabolic risk factors, including obesity (high BMI), hypertension, hypertriglyceridemia, low HDL cholesterolemia, high LDL cholesterolemia, diabetes mellitus, and metabolic syndrome, whereas there was no significant gender difference in the association between serum urate and visceral obesity evaluated by WHtR. The cutoff values for hyperuricemia in men and women from the viewpoint of its association with cardiometabolic risk are lower than the currently used cutoff value and need to be redefined.

## Data Availability

The data that support the findings of this study are available from the corresponding author (I.W.) upon reasonable request.

## Abbreviations Used

AUC: area under the curve
BMI: body mass index
CMI: cardiometabolic index
HDL: high-density lipoprotein
LDL: low-density lipoprotein
OR: odds ratio
ROC: receiver operating characteristic
WHtR: waist-to-height ratio
